# Assessment and Translation of the Antibody-in-Lymphocyte Supernatant (ALS) Assay to Improve the Diagnosis of Enteric Fever in Two Controlled Human Infection Models and an Endemic Area of Nepal

**DOI:** 10.3389/fmicb.2017.02031

**Published:** 2017-10-23

**Authors:** Thomas C. Darton, Claire Jones, Sabina Dongol, Merryn Voysey, Christoph J. Blohmke, Rajendra Shrestha, Abhilasha Karkey, Mila Shakya, Amit Arjyal, Claire S. Waddington, Malick Gibani, Michael J. Carter, Buddha Basnyat, Stephen Baker, Andrew J. Pollard

**Affiliations:** ^1^Oxford Vaccine Group, Centre for Clinical Vaccinology and Tropical Medicine, Department of Paediatrics, University of Oxford, National Institute for Health Research Oxford Biomedical Research Centre, Oxford, United Kingdom; ^2^Wellcome Trust Major Overseas Programme, Hospital for Tropical Diseases, Oxford University Clinical Research Unit, Ho Chi Minh City, Vietnam; ^3^Department of Infection, Immunity and Cardiovascular Disease, University of Sheffield, Sheffield, United Kingdom; ^4^Oxford University Clinical Research Unit, Patan Academy of Health Sciences, Kathmandu, Nepal; ^5^Nuffield Department of Primary Care Health Sciences, University of Oxford, Oxford, United Kingdom

**Keywords:** enteric fever, *Salmonella* Typhi, *Salmonella* Paratyphi A, diagnostic test, antibody-in-lymphocyte supernatant assay, febrile illness, resource-limited settings, bacteremia

## Abstract

New diagnostic tests for enteric fever are urgently needed to assist with timely antimicrobial treatment of patients and to measure the efficacy of prevention measures such as vaccination. In a novel translational approach, here we use two recently developed controlled human infection models (CHIM) of enteric fever to evaluate an antibody-in-lymphocyte supernatant (ALS) assay, which can detect recent IgA antibody production by circulating B cells in *ex vivo* mononuclear cell culture. We calculated the discriminative ability of the ALS assay to distinguish diagnosed cases in the two CHIM studies in Oxford, prior to evaluating blood culture-confirmed diagnoses of patients presenting with fever to hospital in an endemic areas of Kathmandu, Nepal. Antibody responses to membrane preparations and lipopolysaccharide provided good sensitivity (>90%) for diagnosing systemic infection after oral challenge with *Salmonella* Typhi or *S*. Paratyphi A. Assay specificity was moderate (~60%) due to imperfect sensitivity of blood culture as the reference standard and likely unrecognized subclinical infection. These findings were augmented through the translation of the assay into the endemic setting in Nepal. Anti-MP IgA responses again exhibited good sensitivity (86%) but poor specificity (51%) for detecting blood culture-confirmed enteric fever cases (ROC AUC 0.79, 95%CI 0.70–0.88). Patients with anti-MP IgA ALS titers in the upper quartile exhibited a clinical syndrome synonymous with enteric fever. While better reference standards are need to assess enteric fever diagnostics, routine use of this ALS assay could be used to rule out infection and has the potential to double the laboratory detection rate of enteric fever in this setting over blood culture alone.

## Introduction

Infections due to *Salmonella enterica* serovars Typhi (*S*. Typhi) and Paratyphi A (*S*. Partayphi A) affect ~27 million individuals each year, although accurate disease burden calculation is hindered by inadequate diagnostics (Buckle et al., 2012; Wain et al., 2015). New approaches are needed to improve these estimates and to allow timely, accurate discrimination of enteric fever from other causes of non-specific febrile disease (Parry et al., 2011; Darton et al., 2014).

The most sensitive and specific assay currently available for the diagnosis of enteric fever infection is culture of bone marrow aspirate (Crump et al., 2015). Blood culture is more commonly used as a reference standard, although is increasingly recognized as a poor comparator for new diagnostic tests due to low sensitivity (Crump et al., 2015; Storey et al., 2015). Causes of this low sensitivity include the low level bacteremia that occurs with the onset of clinical typhoid symptoms and the frequency of pre-hospital antibiotic consumption in endemic areas, which both affect the number of viable bacteria collected in each sample. These factors are partially mitigated by collecting a larger blood volume (~10 mL), although this is frequently impractical especially in younger children (Wain et al., 1998; Darton et al., 2014). Alternative methods detecting the antibody responses using the Widal test, enzyme-linked immunosorbent assays (ELISAs), or rapid diagnostic tests, are largely disappointing due to cross-reactivity, low sensitivity and pre-existing immunity in endemic settings (Parry et al., 2011; Crump et al., 2015).

Due to the lower blood volumes required and the potential to minimize the effect of prior antimicrobial therapy, the confirmation of enteric fever diagnosis by detection of serologic markers is appealing. Additionally, the possibility for multiplexing assays to target diverse Salmonella species along with other bacterial, viral, and parasitic pathogens is a desirable aim for most commercial diagnostic developers (Baker et al., 2010; Chappuis et al., 2013). Among recent efforts to improve the accuracy of serological methods for enteric fever diagnosis has been the development of an immunodiagnostic test utilizing antibody-in-lymphocyte supernatant (ALS) (Chang and Sack, 2001). Exposure of Salmonella spp. to antigen presenting cells in the gut mucosa generates gut-homing plasma B cells that can be detected *ex vivo* in peripheral blood during cell re-circulation in the reticuloendothelial system (Kirkpatrick et al., 2005; Sheikh et al., 2009). Specifically detecting plasma cell responses precludes the detection of pre-existing immunity, which is common in endemic settings and confounds most other serological approaches. It is therefore possible to detect immunological responses to recent enteric bacterial exposure by assaying ALS secreted by peripheral blood mononuclear cells (PBMC).

Previous studies in Bangladesh have evaluated ALS responses to *S*. Typhi specific-lipopolysaccharide (LPS), formalin inactivated whole cell preparations, and Ty21a membrane preparation (MP) in patients with suspected enteric fever (Sheikh et al., 2009). While IgA and IgG ALS responses were observed against LPS and whole cell preparations, an IgA response only was observed against MP (“anti-MP IgA”), indicating possible recent gut mucosa exposure specifically to *S*. Typhi or *S*. Paratyphi A (Sheikh et al., 2009). Further clinical evaluation suggested that anti-MP IgA responses measured in confirmed typhoid/paratyphoid patients were 100% sensitive and 78–97% specific and short-lived, indicating recent acute illness rather than background cross-reactivity (Khanam et al., 2013; Islam et al., 2016). In this prospective study we aimed to evaluate the diagnostic performance of anti-MP IgA responses using ALS samples collected during two human enteric fever challenge models (*S*. Typhi and *S*. Paratyphi A) (Waddington et al., 2014; McCullagh et al., 2015), and in an endemic enteric fever area of Kathmandu, Nepal (Karkey et al., 2010, 2013, 2016).

## Materials and methods

### Ethics statement

All studies were carried out in accordance with the relevant clinical trial protocols, and the International Conference on Harmonization (ICH) Good Clinical Practice standards. All study participants (or their parents if aged under 18 years in Nepal) gave written informed consent prior to enrolment, in accordance with the Declaration of Helsinki. Illiterate signatories were read the details of the consent form in the presence of a literate witness who could attest to the accurate reading of the consent and the agreement of the signatory (Nepal only). Study protocols for the typhoid and paratyphoid CHIMs were approved by the UK National Research Ethics Service (typhoid challenge, 10/H0604/53; paratyphoid challenge, 14/SC/0004; both Oxfordshire Research Ethics Committee A) and for the Nepal field study by the Nepal Health Research Council (ref. 736) and the Oxford Tropical Ethics Committee (OxTREC, ref. 38-11).

### Human challenge studies

**Figure 1:**
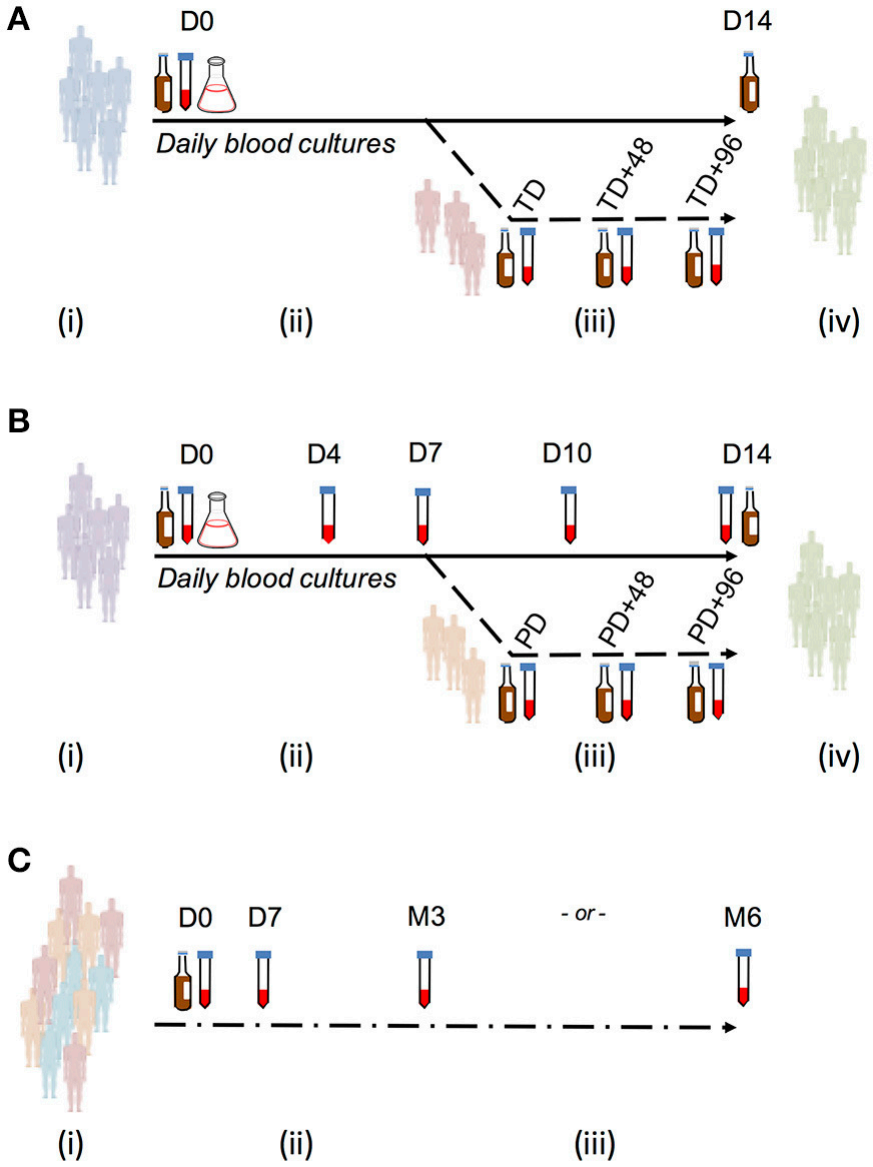
Study sample collection for use in evaluating ALS responses as a diagnostic test for enteric fever. **(A)** Typhoid challenge study (OVG2009/10). Groups of 20 healthy, screened, and enrolled adult participants were challenged with either 1–5 × 10^3^ CFU or 10–50 × 10^3^ CFU *S*. Typhi Quailes strain in a dose-escalation study. Participants were challenged by oral ingestion at baseline (D0) and monitored by daily clinical review, routine blood chemistry and hematology and blood culture. Typhoid diagnosis (TD) was made based on *a priori* definitions of having a temperature ≥38°C for ≥12 h (temperature criterion) and/or having a positive blood culture with *S*. Typhi (blood culture criterion). Samples for measurement of ALS responses were collected at baseline, TD, TD+48 h, and TD+96 h. Antibiotic treatment was initiated at TD or at D14 in all participants not reaching the typhoid diagnosis endpoint. **(B)** Paratyphoid challenge study (OVG2013/07). Groups of 20 healthy, screened and enrolled adult participants were challenged with either 1–5 × 10^3^CFU or 0.5–1 × 10^3^CFU *S*. Paratyphi A strain NVGH308 in a dose-reduction study. Participants were challenged by oral ingestion at baseline (D0) and monitored by daily clinical review, routine blood chemistry, and hematology and blood culture. Paratyphoid diagnosis (PD) was made based on *a priori* definitions of having a temperature ≥38°C for ≥12 h (temperature criterion) and/or having a positive blood culture with *S*. Paratyphi (blood culture criterion). Samples for measurement of ALS responses were collected at baseline, D4, D7, D10, D14, and D28. Alternatively, in those reaching PD, samples were collected instead at PD, PD+48 h, and PD+96 h. Antibiotic treatment was initiated at PD or at D14 in all participants not reaching the paratyphoid diagnosis endpoint. **(C)** Nepal field evaluation. Participants were recruited from individuals presenting with fever and an illness clinically compatible with enteric fever to either Patan Hospital or the Civil Hospital in Patan. Blood for culture and extraction of PBMC/ALS was collected at presentation to hospital (D0), after which antibiotic treatment for enteric fever was given. Further samples were collected to measure ALS responses at D7, month 3 (M3), or month 6 (M6).

Groups of healthy adult volunteers were challenged with a single oral dose of either *S*. Typhi (Quailes strain) or *S*. Paratyphi A (strain NVGH308) at a single site in Oxford, United Kingdom, as previously described (Figure 1; Waddington et al., 2014; McCullagh et al., 2015). Briefly, participants were monitored for 14 days after oral challenge (“baseline”), at 28 days and further time points thereafter. Typhoid/paratyphoid diagnosis and the initiation of antimicrobial treatment was performed if either clinical (temperature ≥ 38°C for ≥12 h) or microbiological (bacteremia) endpoints were reached, and at day 14 in all remaining participants.

Blood for culture (10 mL) was collected each day until day 14 or 96 h after typhoid diagnosis (TD) or paratyphoid diagnosis (PD) was made (TD+96 h or PD+96 h), whichever was the latter; at the TD/PD+0 h time point, 5 mL was collected. Blood culture was performed using continuous automated culture (BACTEC FX system, BD), using standard methods (Health Protection Agency, 2013; Waddington et al., 2014). Duration of bacteremia was calculated as time from first culture positive to first culture negative sample collected. ALS material was collected from a subset of participants diagnosed with typhoid, whereas in the subsequent paratyphoid challenge study, ALS material was collected from all challenge study participants whether diagnosed with paratyphoid fever or not (Figure 1).

### Endemic enteric fever setting

In Kathmandu, Nepal, blood samples were collected prospectively at Patan Hospital in Lalitpur Sub-Metropolitan City (LSMC), and at the Civil Service Hospital both in the Kathmandu Valley area of Nepal. Consenting patients aged >14 years with a clinical diagnosis of typhoid fever (including a history of fever for ≥3 days without a focus of infection) were enrolled. Blood samples (13 mL) were collected at presentation, 7 days and 3 or 6 months later (Figure 1C). Routine blood culture was performed at presentation using local, non-automated culture methods; ALS assays were performed at each time point. ALS results were not used for clinical decision-making. Blood culture, isolation of *Salmonella* serovars and susceptibility testing was performed according to previously described local procedures (Arjyal et al., 2016).

### Antibody-in-lymphocyte supernatant assay

Isolation of PBMC and harvesting of cell culture supernatant was performed in Oxford and at Patan Hospital in-keeping with previously described methods (Sheikh et al., 2009; Khanam et al., 2013; Islam et al., 2016). In brief, PBMC separated from venous blood (5 mL) by density-gradient centrifugation were re-suspended to a concentration of 1 × 10^7^ cells/mm3 before incubation at 37°C, 5% CO_2_ in tissue culture plate wells. After 48 h, culture supernatants were harvested and stored with protease inhibitors at −80°C until further use. Specific IgA and IgM isotype ALS responses to *S*. Typhi LPS (L2387; Sigma, Dorset), *S*. Typhi flagellin (prepared in-house by shearing, centrifugation and purification of a whole cell preparation), *S*. Paratyphi A LPS (O:2; GVGH, Italy), or membrane preparations [made in-house using the method by Sheikh et al. (2009), from *S*. Typhi strain Ty21a, “MP,” or *S*. Paratyphi A NVGH308, “MPN”] were measured by ELISA at dilutions of 1:4 or 1:2, as previously described (Sheikh et al., 2009), using goat anti-human IgA or IgM conjugated to horseradish peroxidase (Biorad, UK). ELISA readouts from each site were standardized; optical density readings were divided by that of a pooled positive control serum standard, multiplied by 100 and expressed as ELISA units (Sheikh et al., 2009; Khanam et al., 2013).

### Statistical analysis

ALS responses were log_10_-transformed for normalization prior to analysis. Analysis of log-transformed ALS assay responses from repeated sampling in challenge studies was evaluated using mixed effects models and adjusted for challenge dose received. Characteristics associated with maximal ALS responses were investigated using multiple linear regression. The anti-log of the parameter estimates from both mixed effects and linear regression models are presented as geometric means with 95% confidence intervals (95% CI).

As no gold-standard test for enteric fever diagnosis is available, the following assumptions were made in determining the sample size for the ALS evaluation in Nepal: (i) that the true prevalence of typhoid in febrile individuals enrolled to the study was 80%, and (ii) that blood culture would correctly classify 50% of participants as having enteric fever. Thus in a sample size of 100, there would be 80% power to detect a difference in the proportion diagnosed through blood culture and those through ALS, using McNemar's test and assuming a discordant pairs rate of 20% and a 15% rate of non-evaluable samples.

We determined an *a priori* cut-off threshold for a positive ALS response by taking the geometric mean of all baseline samples (or in Nepal the month 3 or 6 result) plus three standard deviations. Assay performance was analyzed by receiver-operator characteristic (ROC) curves using the pROC package in R (version 3.3.0; Robin et al., 2011), and inter-assay agreement by the Bland-Altman method (Giavarina, 2015), implemented using the MethComp package (version 1.22; Carstensen et al., 2013).

## Results

### ALS assay in a *Salmonella* typhi challenge study

In the controlled human infection model of typhoid fever, 21 participants ingested 1–5 × 10^3^ CFU and 20 ingested 10–50 × 10^3^ CFU of *S*. Typhi, resulting in attack rates of 55% (11/20) and 65% (13/20), respectively (Waddington et al., 2014). ALS material was collected from 11/11 and 12/13 of these participants developing typhoid infection, of which 9/11 (82%) and 10/12 (83%) were confirmed by positive blood culture (Table 1).

**Table 1:** Oxford challenge studies: clinical parameters.

**Study**	**Typhoid challenge (*****N*** = **23)**		**Paratyphoid challenge (*****N*** = **40)**	
Challenge agent, strain	*S. enterica* serovar Typhi, Quailes strain		*S. enterica* serovar Paratyphi A, strain NVGH308	
Participants included in ALS analysis (type)	Challenge and diagnosed participants only		All challenged participants	
Challenge dose level	Low	High	Low	Lower
Target dose	1–5 × 10^3^	10–50 × 10^3^	1–5 × 10^3^	0.5–1.0 × 10^3^
Actual dose, median [range]	1.05 × 10^3^ [0.7–1.8]	19.8 × 10^3^ [15.5–27.0]	2.40 × 10^3^ [2.2–2.8]	0.91 × 10^3^ [0.7–1.3]
**Number assessed**	**11**	**12**	**20**	**20**
Male sex, *N* (%)	11 (100)	7 (58)	10 (50)	11 (55)
Median age, yrs [range]	27 [19–41]	28 [20–44]	28 [19–49]	28 [23–50]
Attack rate, *N* (%)	na	na	12 (60)	8 (40)
Median time to diagnosis, days [IQR]	11 [7–13]	8 [6–9]	6 [6–7]	8 [8–9]
**Mode diagnosis**, ***n*****(%)**				
Temperature criterion	6 (60)	7 (58)	1 (9)	0 (0)
Blood culture criterion	4 (40)^*^	5 (42)	11 (81)	8 (100)
≥1 positive blood cultures, N (% diagnosed)	9 (90)^*^	10 (83)	12 (100)	8 (100)
Median duration bacteraemia, days [IQR]	1 [1–2]	2 [1–3]	4 [2.5–5]	1 [1–4.5]
Median temperature at diagnosis, °C [IQR]	37.9 [36.7–38.7]	37.8 [37.3–38.1]	37.4 [36.9–38.0]	36.9 [36.5–37.3]
Median heart rate at diagnosis, bpm [IQR]	96 [79–109]	95 [83–104]	87 [80–92]	83 [79–89]
Typhoid symptom triad^*^, *N* (%)	11 (55)	12 (75)	6 (30)	3 (15)

* *Typhoid symptom triad = fever, headache plus abdominal pain. na, not applicable*.

An assessment of anti-MP IgA ALS responses in participants developing typhoid infection demonstrated a significant increase between baseline and TD time points (baseline to all time points *p* < 0.0001; Table 2). These changes were consistent across all time points until TD+96 h (*p* = 0.860), and were observed in most participants (Figure S1). IgA ALS responses against *S*. Typhi-specific LPS were comparable to those against MP, however responses against flagellin were less pronounced (Table 2, Figure S1). Maximal anti-MP IgA ALS responses were significantly associated with bacteremia duration (Spearman's ρ = 0.42, *p* = 0.048) and age (Spearman's ρ = −0.44, *p* = 0.038). In multiple linear regression models, these responses were significantly lower in older participants (geometric mean ratio, GMR 0.95 per year, 95% CI 0.91–0.99, *p* = 0.023) and higher in those with longer durations of bacteremia (GMR 1.45 per day bacteremia, 95% CI 1.07–1.96, *p* = 0.020).

**Table 2:** Analysis of ALS responses to three antigens (MP, LPS, and flagellin) in participants challenged with ST (baseline) and diagnosed with typhoid infection.

	**Geometric mean (95% CI)**	Fold change from baseline^*^ (95% CI)	p^*^	***p* (Type III)^#^**
**MP**				
Baseline	5.3 (4.9–5.8)			
TD	27.3 (16.5–45.3)	5.37 (3.21–8.99)	<0.0001	0.860
TD+48 h	23.5 (14.8–37.4)	4.52 (2.9–7.06)	<0.0001	
TD+96 h	27.7 (16.8–45.7)	5.22 (3.22–8.47)	<0.0001	
**LPS**				
Baseline	6.2 (5.7–6.7)			
TD	29.8 (18.0–49.3)	5.16 (3.09–8.6)	<0.0001	0.873
TD+48 h	26.3 (16.7–41.4)	4.41 (2.83–6.87)	<0.0001	
TD+96 h	31.0 (18.8–50.9)	5.06 (3.13–8.18)	<0.0001	
**FLAGELLIN**				
Baseline	11.7 (10.8–12.7)			
TD	15.8 (12.1–20.6)	1.39 (1.13–1.72)	0.003	0.248
TD+48 h	15.2 (13.1–17.7)	1.31 (1.09–1.57)	0.005	
TD+96 h	12.7 (10.9–14.7)	1.11 (0.91–1.35)	0.304	

*Output are from mixed effects models with a random intercept for each participant. Variables for dose and visit included in the model as fixed effects*.

* *Adjusted for challenge dose received (high or low)*.

# *P-value testing whether change from baseline is different across visits. TD, day of typhoid diagnosis*.

**Figure 2:**
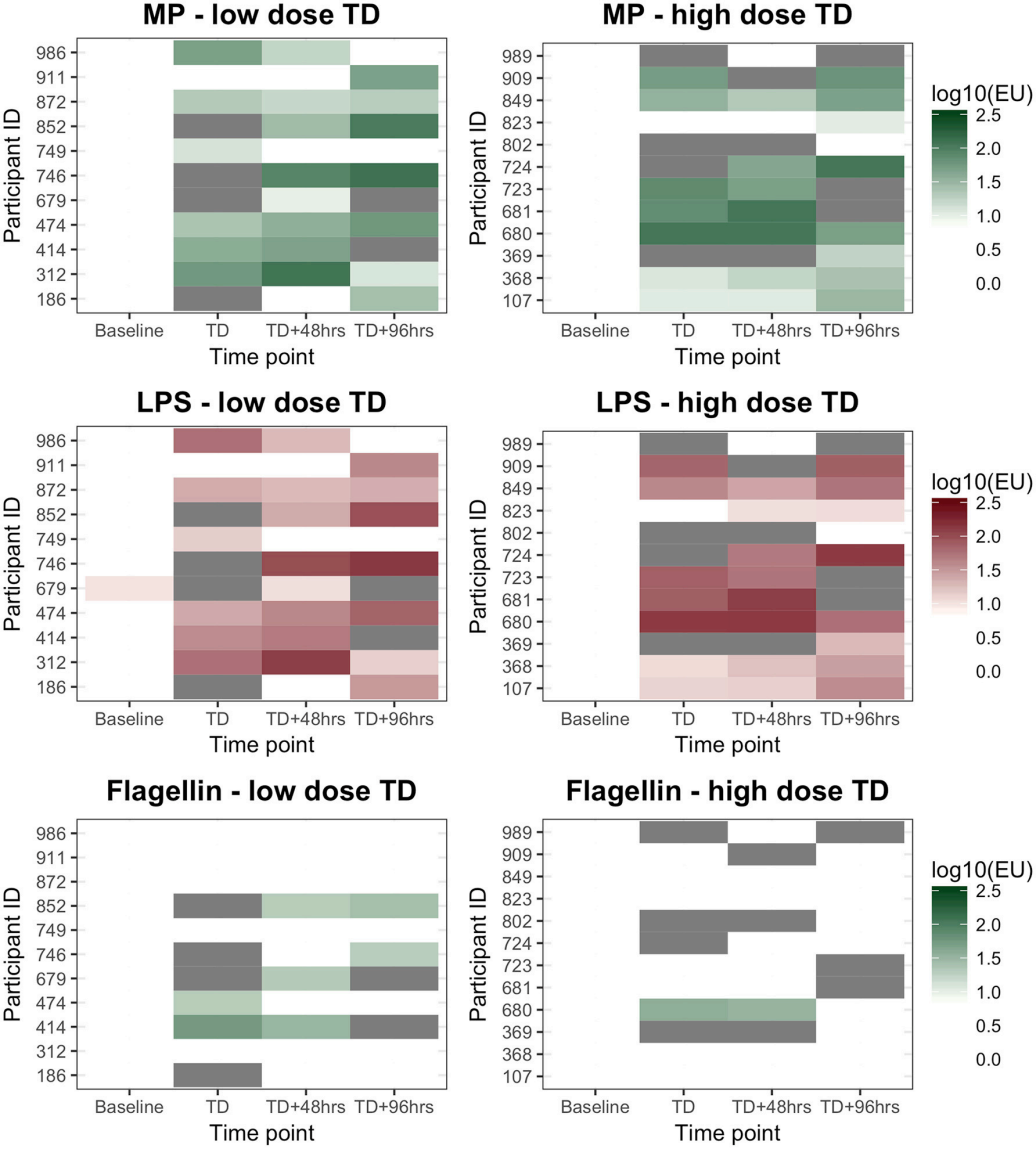
ALS IgA diagnostic responses to MP, LPS, and flagellin in participants diagnosed with typhoid after challenge. Low dose, 1–5 × 10^3^CFU; High dose, 10–50 × 10^3^CFU. “*White*” indicates the titer was below the diagnostic cut-off (log_10_0.95, log_10_1.00, and log_10_1.28EUs for the three antigens tested, respectively). “*Gray*” indicates sample not available.

Anti-MP IgA ALS responses diagnostic of typhoid infection were recorded in 11/11 (100%) and 10/12 (83%) participants who developed typhoid infection following challenge with 10^3^ or 10^4^ CFU *S*. Typhi, respectively (Figure 2). With the study definition of TD as the reference standard, the sensitivity of anti-IgA MP ALS responses was 91% (95%CI 72–99%). ALS IgA responses against LPS correlated with MP responses (*r* 0.99, *p* < 0.0001), were completely concordant with the diagnostic classification (Table S1), and had acceptable inter-assay agreement (Figure S2). In contrast, while ALS IgA responses against flagellin correlated with MP responses, they demonstrated poor agreement and a significant bias toward lower responses in the flagellin assay (Figure S2). Fewer participants had flagellin IgA titers exceeding the diagnostic threshold at time points at and after TD (Figure 2).

### ALS assay in a *Salmonella* paratyphi a challenge study

In a *S*. Paratyphi A challenge study, 40 participants ingested either 1–5 × 10^3^ CFU or 0.5–1 × 10^3^ CFU of *S*. Paratyphi A, resulting in attack rates of 12/20 (60%) and 8/20 (40%), respectively (Table 1). All paratyphoid diagnoses were confirmed by positive blood cultures and ALS samples were collected at baseline and at days 4, 7, 10, 14, and 28 from all participants, in addition to other time points after paratyphoid diagnosis (PD, PD+48 h, and PD+96 h; Figure 1).

Significant increases in ALS IgA responses against *S*. Paratyphi A MP (MPN) were observed at PD time points in diagnosed participants (all *p* < 0.0001) and did not change at later PD time points (*p* = 0.072; Table 3). Maximal anti-MPN IgA ALS responses against MP positively correlated with challenge dose (Pearsons's ρ = 0.33, *p* = 0.035), maximal oral temperature (Spearman's ρ = 0.43, *p* = 0.005), duration of bacteremia (Spearman's ρ = 0.65, *p* < 0.0001), and time elapsed between challenge and diagnosis (PD only, Spearman's ρ = −0.45, *p* = 0.046). In a multiple linear regression model, the duration of bacteremia was the only variable significantly associated with maximal anti-MPN IgA ALS responses (GMR 1.25 per day bacteremia, 95%CI 1.11–1.39, *p* < 0.0001).

**Table 3:** Analysis of ALS responses to MPN and LPS in participants challenged with *Salmonella* Paratyphi A and diagnosed with paratyphoid infection.

	**Geometric mean (95% CI)**	Fold change from baseline^*^ (95% CI)	p^*^	***p* (Type III)^#^**
**MP**				
Baseline	7.3 (7.0, 7.6)			
PD	19.2 (13.5, 27.2)	2.54 (1.76, 3.66)	<0.0001	0.072
PD+48 h	17.3 (10.9, 27.4)	2.29 (1.59, 3.3)	<0.0001	
PD+96 h	30.5 (20.1, 46.1)	4.03 (2.8, 5.81)	<0.0001	
**LPS**				
Baseline	7.2 (6.9, 7.5)			
PD	15.2 (10.3, 22.5)	2.07 (1.4, 3.06)	<0.0001	0.085
PD+48 h	16.7 (10.8, 25.8)	2.28 (1.54, 3.36)	<0.0001	
PD+96 h	27.1 (17.0, 41.0)	3.7 (2.51, 5.46)	<0.0001	

*Output are from mixed effects models with a random intercept for each participant. Variables for dose and visit included in the model as fixed effects*.

* *Adjusted for challenge dose received (high or low)*.

# *P-value testing whether change from baseline is different across visits. PD, Day of paratyphoid diagnosis*.

**Figure 3:**
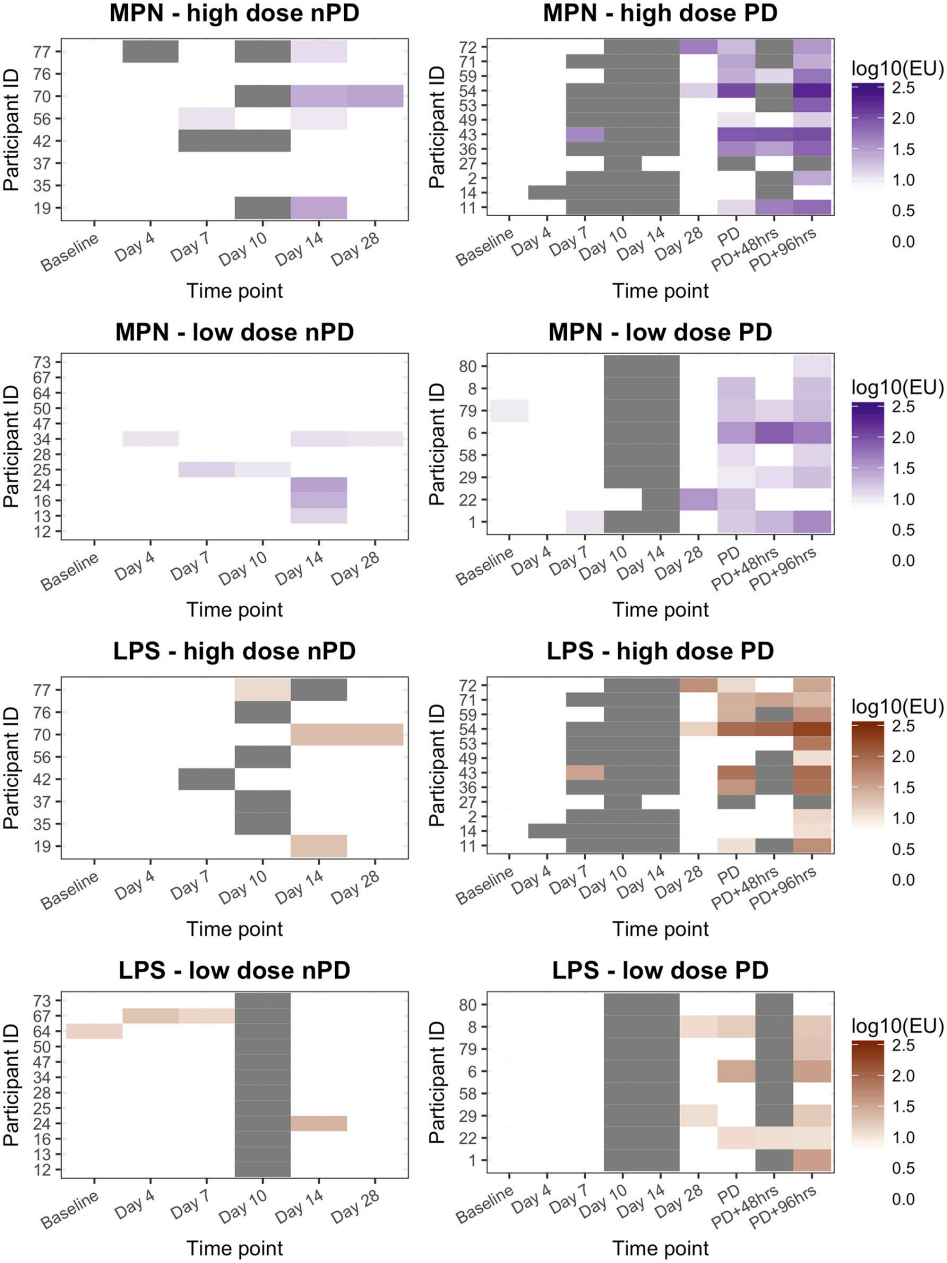
ALS IgA responses to MPN or LPS in participants challenged with *Salmonella* Paratyphi A according to challenge dose level and challenge outcome. High dose, 1–5 × 10^3^ CFU; Low dose, 0.5–1 × 10^3^ CFU. nPD, paratyphoid not diagnosed; PD, paratyphoid diagnosed. “*White”* indicates the titer was below the diagnostic cut-off (10EU). “*Gray”* indicates sample not available.

In this study, anti-MPN IgA ALS responses diagnostic of paratyphoid infection were recorded in 10/12 (83%) participants and 8/8 (100%) participants who developed paratyphoid infection following challenge with 1–5 × 10^3^ or 0.5–1 × 10^3^ CFU of *S*. Paratyphi A, respectively (Figure 3). This resulted in a sensitivity of 90% (95%CI 68–99) and specificity of 60% (95%CI 36–81) for the detection of the study-defined PD endpoint (Table 4). Eight participants had diagnostic anti-MPN IgA ALS responses at day 14 without reaching PD (Figure S3). These participants were notably more symptomatic, for example, reporting more severe (Grade 3) symptoms than other nPD individuals (*p* = 0.014 Mann–Whitney *U*-test).

**Table 4:** Performance of anti-MPN IgA ALS responses as a serodiagnostic test compared to the study paratyphoid diagnosis definition (fever and/or bacteremia).

		**Study paratyphoid diagnosis (%)**		
		**Yes**	**No**	**Total**
MPN diagnostic response, *n* > 9.9EU (%)	Yes	18 (45)	8 (20)	26 (60)
	No	2 (5)	12 (45)	14 (40)
	Total	20 (50)	20 (50)	40 (100)
		**LCL**	**UCL**	
Sensitivity	90.0%	68.3%	98.8%	
Specificity	60.0%	36.1%	80.9%	
PPV	69.2%	48.2%	85.7%	
NPV	85.7%	57.2%	98.2%	

*PPV, positive predictive value; NPV, negative predictive value. LCL, lower confidence limit; UCL, upper confidence limit*.

Overall, there was good agreement between the anti-MPN and anti-LPS IgA ALS assays (Figure S4). As with the false-positive responses identified in the anti-MPN assay, positive anti-LPS IgA ALS responses were also observed in individuals without PD (Figure 3 and Table S2). The AUC ROC for anti-MPN IgA ALS responses to detect participants meeting the study endpoint definition for PD (reference standard) was 0.85 (Figure S5). The AUC for the anti-LPS IgA assay responses was not significantly different (AUC 0.80, 95% CI 0.66–0.94, *p* = 0.255; Figure S5).

### ALS assay in a febrile disease cohort in Kathmandu

Between June 2013 and December 2014, 177 patients presenting with non-specific febrile illness were recruited into a prospective diagnostics study in Nepal (Figure S6). Table 5 describes the patient cohort (see also Table S3). Blood cultures confirmed 21/177 (12%) had *S*. Typhi and 14/177 (8%) had *S*. Paratyphi A bacteremia. Blood for ALS measurements was collected at day 0, day 7, and at months 3 or 6 (Figure 1). Anti-MP IgA responses were measured at day 0 for 173/177 patients (Figure S6).

**Table 5:** Nepal ALS study participant demographics and clinical characteristics.

**Blood culture result**	***S*****. Typhi**		***S***. **Paratyphi A**		**No growth**		***p^#^***
**Number**	**21**		**14**		**142**		
**Demographic**	***n***^A^	**Result**	***n***	**Result**	***n***	**Result**	
Age, median (IQR)	21	26 (17–30)	14	20 (18–26)	142	25 (19–32)	0.179
Male sex (%)	21	17 (81)	14	11 (79)	142	93 (66)	0.565
Residence (%)	19		10		130		
Lalitpur		8 (42)		4 (40)		83 (64)	
Kathmandu		8 (42)		4 (40)		32 (25)	
Bhaktapur		1 (5)		1 (10)		6 (5)	
Other^B^		2 (11)		1 (10)		9 (7)	
Median residence Kathmandu Valley, yrs (IQR)	6	3 (2–4)	7	2 (2–6)	60	10 (7–19)	**<0.0001**
**WATER SUPPLY**							
Water source (%)	19		11		137		
Supply tap		10 (53)		3 (27)		89 (65)	
Jar		3 (16)		7 (64)		18 (13)	
Water tanker		4 (21)		0		13 (9)	
Well		1 (5)		0		9 (7)	
Stone spout		1 (5)		1 (9)		8 (6)	
Other		0		0		4 (3)	
Use water treatment^C^ (%)	19	9 (11.0)	11	4 (4.9)	139	69 (84.1)	0.694
**CLINICAL CHARACTERISTICS**							
Mean illness duration (range), days	18	5.7 (2–15)	10	6.2 (3–14)	138	5.5 (2–20)	0.425
Prior antibiotics given, *n* (%)	9	6 (67)	3	3 (100)	91	39 (43)	0.067
Median prior antibiotic duration (IQR), days	4	3 (1–3)	2	3 (1–4)	37	3 (2–3)	0.778
Previous typhoid, *n* (%)	17	2 (12)	10	2 (20)	137	43 (31)	0.198
Previous typhoid vaccine, *n* (%)	18	0	10	0	138	2^*^ (1)	0.814
Family member with typhoid, *n* (%)	18	3 (17)	11	3 (27)	136	17 (13)	0.372

A *n, number for which clinical data were available*.

B *Other includes Dhading 2/159, Kavre 3/159, Other 7/159*.

C *Water treatment, including “filtering” 51/169, “boiling” 24/169, “chlorination” 3/169, “other” 4/169. No growth includes contaminants N = 8/177 (4.5%)*.

* *Both received vaccine within 3 years prior to presentation*.

# *Group-wise comparisons were made by Pearson's chi-squared or Kruskal–Wallis tests. The p value in bold indicates that it reaches significance at the 0.05 threshold*.

**Figure 4:**
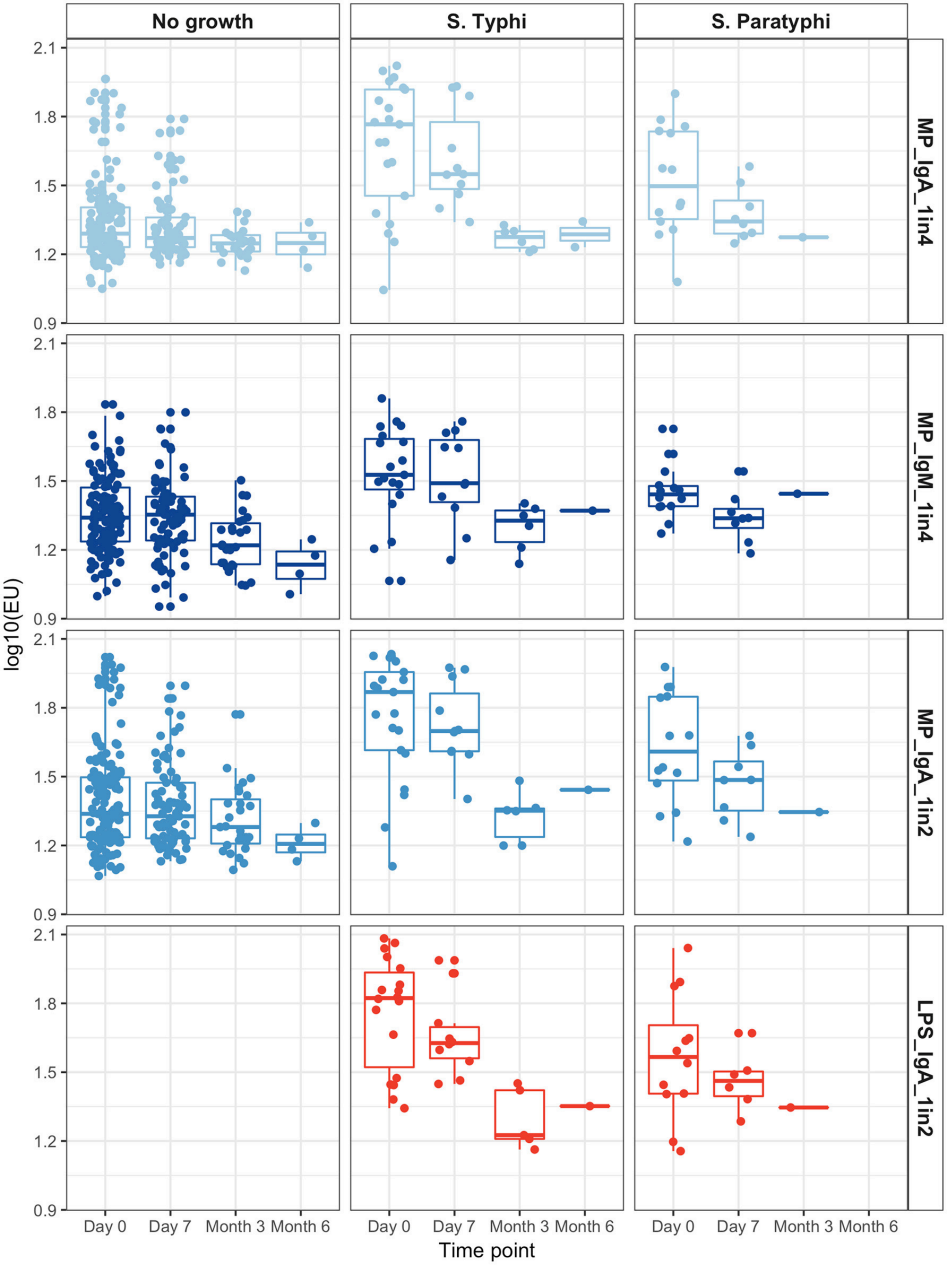
Group ALS responses in each assay condition by time point, according to blood culture result.

Overall, anti-MP IgA ALS responses in samples collected on day of presentation (day 0) were significantly higher in bacteremic patients compared with those with a sterile blood culture (GMR bacteremia vs. no growth 1.85, 95%CI 1.55–2.21; *p* < 0.0001). Further, anti-MP IgA ALS responses at day 0 were significantly higher in *S*. Typhi and *S*. Paratyphi A culture positive patients compared to with those with a sterile blood culture (GMR 2.13, 95%CI 1.72–2.64, *p* < 0.0001 for *S*. Typhi; and 1.50, 1.16–1.94, *p* = 0.002 for *S*. Paratyphi A; Figure 4). There were significant differences in ALS measurements between day 0 and 7 in all three groups, including a 9% decrease in the no growth group (GMR day 7/day 0: 0.91, 95%CI 0.84–0.98, *p* = 0.018). Larger decreases of 32 and 39% were observed in the *S*. Typhi and *S*. Paratyphi bacteremic patients, respectively (*p* < 0.001, Figure 4).

Data from convalescent samples at month 3 and month 6 were combined. Anti-MP IgA ALS responses were significantly lower at month 3/6 than day 0 in those with a positive blood culture (*p* < 0.0001). In contrast, no significant change in ALS response was observed in patients with sterile blood cultures (*p* = 0.202, Figure 4 and Figure S7).

### ALS assay diagnostic performance

Using blood culture-confirmed cases as the reference standard and applying a diagnostic threshold cut-off of 28.8EU (derived as described in Methods), anti-MP IgA ALS responses resulted in a test sensitivity of 85.7% (95% CI 69.7%−95.2%) and specificity of 51.4% (42.8–60.0%, Table 6). Further exploration of these responses by ROC curve analysis demonstrated an AUC of 0.79 (Figure S9), indicating “fair” to “good” test accuracy (Metz, 1978). ALS IgA MP responses using the 1:2 or 1:4 dilution resulted in comparable ROC curves (*p* = 0.097) (Figure S8). There was also was no significant difference in AUC values for MP IgM compared to MP IgA 1:4 (*p* = 0.357).

**Table 6:** Contingency table of Nepal field study anti-MP IgA ALS results (1:4 concentration) and blood-culture confirmed cases.

		**Bacteremia (%)**		
		**Yes**	**No**	**Total**
ALS IgA MP 1:4 diagnostic response, n > 28.8EU (%)	Yes	30 (17.3)	67 (38.8)	97 (56.1)
	No	5 (2.9)	71 (41.0)	76 (43.9)
	Total	35 (20.2)	138 (79.8)	173
		**LCL**	**UCL**	
Sensitivity	85.7%	69.7%	95.2%	
Specificity	51.4%	42.8%	60.0%	
PPV	30.9%	21.9%	41.1%	
NPV	93.4%	85.3%	97.8%	

While ALS demonstrated reasonable sensitivity, measured specificity using blood culture confirmed cases as the reference standard was poor. Amongst all patients and using a threshold of 28.8EU for anti-MP IgA ALS, 72/177 (41%) participants had discordant blood culture and ALS responses. Five (6.9%) of these participants were BC+/ALS- and 67 (63.1%) participants were BC−/ALS+ (*p* < 0.0001 McNemar's test). To further investigate this, participants were classified as “low” responders if anti-MP IgA ALS responses were <25th percentile (<1.39 log_10_EU) and “high” responders if >75th percentile (>1.64 log_10_EU). Of the 79 participants falling into these quartiles, 2/36 (5.6%) in the low and 22/42 (52.3%) in the high responder group had confirmed *S*. Typhi or *S*. Paratyphi bacteremia (Table S4). High responders were significantly more likely to report fever, headache and abdominal pain (*p* = 0.024), and had a significantly lower white cell count (WCC) compared to low responders (*p* < 0.0001).

## Discussion

New diagnostic tests for accurately detecting cases of enteric fever are needed, to enable prompt appropriate treatment of patients with antibiotics and to accurately measure the impact of prevention strategies including vaccination. Here, we aimed to evaluate a promising new ALS assay for use as an enteric fever diagnostic in two recently developed controlled human infection models (CHIM) and in an endemic setting.

Our findings from the human models suggest that detecting IgA responses to homologous membrane preparations in ALS sample material could be a sensitive method for detecting enteric fever cases. As expected, ALS responses to MP and LPS were very similar in both typhoid and paratyphoid infections, suggesting that MP could be used as a suitable reagent in settings were a suitable source of purified LPS is not available. Despite its inclusion as a component of the Widal test, diagnostic responses to flagellin during acute infection were less convincing, at least in the settings studied here. Specificity was less easy to evaluate in the challenge setting, where alternate causes of fever are not anticipated during the brief two-week challenge period. In Nepal, the sensitivity of anti-MP IgA ALS responses to detect bacteremia-confirmed enteric fever cases from patients presenting with non-specific febrile illness was similar to the sensitivity observed in challenge studies, and ROC curve analysis suggested “good” performance as a diagnostic test. Specificity was low when using blood culture as a reference standard in Nepal. This may be related to the low sensitivity of blood culture in this setting, probably driven by frequent exposure to antimicrobials prior to enrolment. With the conservative assumption that those participants with ALS responses >75th percentile were true enteric fever cases, as indicated by the compatible clinical features within this group, additional use of the ALS assay in an endemic setting may double the number of cases confirmed by blood culture alone.

In the CHIM studies we found that the ALS assay, utilizing IgA responses against MP, was ~90% sensitive for detecting challenged participants developing typhoid/paratyphoid infection. Furthermore, these responses persisted for at least 96 h after diagnosis and resolved by day 28. Responses in both challenge studies were strongly associated with the duration of bacteremia, likely increasing the period of antigen presentation and thus amplification of the immune response. In Nepal the sensitivity of the ALS IgA MP (1:4 assay) responses for detecting bacteremia-confirmed enteric fever patients was similar to the challenge studies (85.7%), although lower than those observed in previously published ALS studies which consistently reported sensitivities of 100% (Sheikh et al., 2009; Khanam et al., 2013; Islam et al., 2016). Reasons for higher reported sensitivity in other studies may include younger patients, lower pre-hospitalization antimicrobial treatment, or a greater proportion of female study participants compared with our evaluation (Sheikh et al., 2009; Khanam et al., 2013); all of these factors can influence either the degree of bacteremia or the resulting immune response (Wain et al., 1998; Dewan et al., 2013; Garcia-Gomez et al., 2013). Our finding of <100% sensitivity in Nepal is further supported by the results from the challenge studies, in which 2/23 and 2/40 *S*. Typhi and *S*. Paratyphi A challenged and diagnosed participants, respectively, failed to demonstrate a diagnostic response, despite being bacteremic. While the threshold used was higher than that in previous studies (28.8EU vs. 10EU), this was supported by data from assays performed in Nepal using samples collected from several healthy controls in the UK and in Nepal (~20EU). As recently identified in serological surveys, this suggests that a dynamic cut-off for assay positivity may be required, reflecting background exposure (i.e. seroreactivity) and disease seroprevalence.

Assay specificity was difficult to interpret from the challenge studies; in the typhoid challenge model only diagnosed participants were included making this incalculable. Results from the paratyphoid challenge model suggested a relatively low specificity of ~60%. Notably, the majority of PD participants were diagnosed based on blood culture criteria which, with an assumed ~80% sensitivity, is likely to have resulted in missed case detection. Individuals with diagnostic ALS responses but negative blood cultures were more symptomatic and may have had subclinical or developing infection, which would have been abrogated by antimicrobial therapy on day 14. Additionally, high ALS responses may also reflect a successful immune response to clear *S*. Paratyphi from the intestinal mucosa without clinical disease. Necessary restriction of the challenge period to 14 days is is an important limitation to using this CHIM, especially for evaluating serological responses. Treatment of all participants at day 14 was required to ensure that subclinical infection was not missed and to minimize the risk of complications including bowel perforation which are more frequently seen in after 3-weeks of infection.

In Nepal, assay specificity was also relatively low at 51.4% using the a priori method for calculating the diagnostic cut-off. Reasons for this low measurement are likely to be multifactorial, but strongly influenced by the use of blood-culture confirmed cases as the reference standard. This highlights the inadequacy of using blood culture as the reference/gold standard test with which to evaluate newer methods. Previous methods to overcome this have included use of latent class analysis or a composite diagnostic endpoint made of alternate, less effective tests (Islam et al., 2016). While these methods demonstrate apparently high levels of specificity for the ALS assay, febrile control populations are often comprised of patients with non-enteric diseases that present with features that are unlike acute enteric fever (Khanam et al., 2013; Islam et al., 2016). In addition, previous evaluations of the ALS method have often used the Widal test as a method to screen for a “true negative” population, which removes any participants with false-positive seroreactivity (Islam et al., 2016). Of note, the low specificity of the ALS assay (51.4%) using this cut-off threshold (28.8EU) was equivalent to that found in the previous evaluation of the Widal test in Kathmandu (58 and 51% for the anti-H and anti-O responses, respectively) (Adhikari et al., 2015), but lower than that reported for Tubex TF and Typhidot rapid diagnostic tests, the specificity for which are reported to be between ~60 and 90%, depending on the population studied (Keddy et al., 2011; Thriemer et al., 2013).

The findings of this evaluation are likely applicable to a wide variety of resource-limited settings, and reflect a “real-world” situation in many tropical healthcare settings. The difficulty of the task in accurately detecting patients with enteric fever etiology in our ALS evaluation cohort is demonstrated by comparison with that of the parent treatment study (Arjyal et al., 2016). In terms of performing the ALS assay in other similar facilities in endemic regions, the training of local dedicated staff was uncomplicated and the logistic requirements were relatively minimal. Some basic laboratory infrastructure was required, including an incubator, centrifuge, ELISA plate reader and reagents for performing PBMC separation. On-going work is attempting to address these requirements to make the assay more amenable to widespread use; this includes simplifying the PBMC isolation procedure, removing the need for a specific CO_2_ incubator and using a lateral flow-device rather than ELISA method for antibody detection (Khanam et al., 2013). ALS responses appear to be robust and durable for at least 7 days after hospital presentation, suggesting that only a single sample within this time period is required. While further work is required to detail the resolution of these responses over time, titers appeared to normalize by 3 months after presentation suggesting that they are able to detect acute infection and are not affected by the development of exposure-related immunity.

Our data revealed several useful insights into the likely underlying disease etiology in patients presenting with non-specific febrile disease in an endemic region. It was likely that many of the patients recruited to the study in Nepal had enteric fever as the true underlying cause of their presentation but that due to the constraints of blood culture this was not detected. This was supported by evaluation of the clinical characteristics of high-low responders in which only ~50% of those participants with clinically compatible signs and symptoms and high ALS responses were blood culture confirmed cases. This also suggests that the specificity of the assay is likely to be considerably higher than that estimated by our study; further work is ongoing to determine the true etiological cause of fever in these study participants by excluding other common local febrile illnesses (Thompson et al., 2015). In high burden endemic settings, the high negative predictive value of the assay could be used to exclude enteric fever in febrile individuals presenting to hospital. In settings where the etiology is more likely to be mixed, incorporation of rapid diagnostic tests for malaria and dengue would also need to be considered; Kathmandu has a low incidence of both of these conditions, although rickettsial infections are frequent (Thompson et al., 2015). An alternative strategy could be to investigate the performance of ALS responses to malaria, dengue or rickettsial antigens as has been performed with tuberculosis and pneumococcal infection (Jiao et al., 2015).

In conclusion, we have evaluated and characterized the performance of an ALS assay for the detection of typhoid or paratyphoid infection in two human challenge models and translated and evaluated the diagnostic performance in a high incidence endemic setting. Although not currently adapted for use outside of the clinical research setting in resource-limited settings, the anti-MP IgA ALS assay offers good sensitivity for detecting cases of typhoid/paratyphoid over existing serological approaches and could double enteric fever disease burden estimates over those provided by blood culture data alone. Further work is needed to evaluate alternative, improved reference standards, to optimize antigen selection and to compare ALS responses with other currently available alternative diagnostic methods including the rapid serological diagnostic tests.

## Author contributions

TD and AP designed the studies and acquired the funding. TD, CJ, CB, CW, and MG performed the CHIM studies, acquired the samples, and performed the assays. CJ, SD, RS, MS, AK, and AA transferred the assays and performed sample collection and diagnostic assays in Nepal with oversight and active input from BB and SB. TD, CB, and MV performed the data analysis. The data were curated by SB, AK, CJ, and TD. Manuscript was prepared by TD with support from MV and subsequently reviewed with input by all authors.

### Conflict of interest statement

The authors declare that the research was conducted in the absence of any commercial or financial relationships that could be construed as a potential conflict of interest. The reviewer EB and handling Editor declared their shared affiliation.
