# Supplementary material for: Assessment and Translation of the Antibody-in-Lymphocyte Supernatant (ALS) Assay to Improve the Diagnosis of Enteric Fever in Two Controlled Human Infection Models and an Endemic Area of Nepal
Source: Front Microbiol. 2017 Oct 23;8:2031. doi: 10.3389/fmicb.2017.02031 (PMC5660281; doi:10.3389/fmicb.2017.02031)
Supplement: Supplementary file 1 [file Table1.DOCX]

Supplementary Material

Assessment and translation of the Antibody-in-Lymphocyte Supernatant (ALS) assay to improve the diagnosis of enteric fever in two controlled human infection models and an endemic area of Nepal

Thomas C. Darton^1,2,3*^, Claire Jones^1^, Sabina Dongol^4^, Merryn Voysey^1,5^, Christoph J. Blohmke^1^, Rajendra Shrestha^4^, Abhilasha Karkey^2,4^, Mila Shakya^4^, Amit Arjyal^4^, Claire S. Waddington^1^, Malick Gibani^1^, Michael J. Carter^1,4^, Buddha Basnyat^4^, Stephen Baker^2^, and Andrew J. Pollard^1^

*** Correspondence:** Dr Thomas C. Darton,

[Thomas.darton@paediatrics.ox.ac.uk](mailto:Thomas.darton@paediatrics.ox.ac.uk) / tdarton@oucru.org

# Supplementary Tables

Table S1. Contingency table comparing diagnostic ALS IgA MP responses with LPS for diagnosis of typhoid infection.

|  |  | **LPS diagnostic response,**  **n>10EU (%)** | | |
| --- | --- | --- | --- | --- |
|  |  | **No** | **Yes** | **Total** |
| **MP diagnostic response, n>9EU (%)** | **No** | 3 (13) | 0 (0) | 3 (13.04) |
|  | **Yes** | 0 (0) | 20 (87) | 20 (87) |
|  | **Total** | 3 (13) | 20 (87) | 23 (100) |

Table S2. Contingency table comparing diagnostic ALS IgA MPN responses with LPS for diagnosis of paratyphoid infection.

|  |  | **LPS diagnostic response,**  **n>10.9EU (%)** | | |
| --- | --- | --- | --- | --- |
|  |  | **Yes** | **No** | **Total** |
| **MPN diagnostic response, n>9.9EU (%)** | **Yes** | 18 (45) | 1 (2.5) | 19 (47.5) |
|  | **No** | 6 (15) | 15 (37.5) | 21 (52.5) |
|  | **Total** | 24 (60) | 16 (40) | 40 (100) |

Table S3. Symptoms reported by patients recruited to ALS study in Nepal.

| **Blood culture result** | ***S*. Typhi** | ***S.* Paratyphi** | **No growth** | ***p^#^*** |
| --- | --- | --- | --- | --- |
| Number | 21 | 14 | 142 |  |
| **Symptom** | n/N (%) | n/N (%) | n/N (%) |  |
| Fever | 16/16  (100) | 7/7  (100) | 51/51  (100) | *NA* |
| Headache | 15/16  (93.8) | 7/7  (100) | 45/51  (88.2) | 0.538 |
| Abdominal pain | 9/16  (56.3) | 2/7  (28.6) | 12/51  (23.5) | **0.047** |
| Anorexia | 11/16  (68.8) | 4/7  (57.1) | 26/51  (51) | 0.457 |
| Diarrhoea | 10/16  (62.5) | 2/7  (28.6) | 11/52  (21.2) | **0.007** |
| Constipation | 1/16  (6.3) | 1/7  (14.3) | 1/52  (1.9) | 0.256 |
| Vomiting | 5/16  (31.3) | 0/7  (0) | 10/51  (19.6) | 0.225 |
| Cough | 6/16  (37.5) | 3/7  (42.9) | 14/52  (26.9) | 0.553 |
| Myalgia | 2/16  (12.5) | 3/7  (42.9) | 10/51  (19.6) | 0.244 |

^#^*p* value testing for group-wise differences (Pearson’s chi-squared test). N, number for whom data were available.

**Table S4. Characteristics of Low and High ALS IgA MP responders.** * Typhoid triad = fever, headache and abdominal pain

|  | **Low ALS IgA MP responder**  **<1.39 log_10_ EU** | **High ALS IgA MP responder**  **>1.64 log_10_ EU** | ***p*** |
| --- | --- | --- | --- |
| Number | 36 | 42 |  |
| Median ALS IgA MP (IQR), log EU | 1.34  (1.32–1.38) | 1.93  (1.76–2.04) | **<0.0001** |
| Bacteraemia, n (%) | 2 (5.6) | 22 (51.2) | **<0.0001** |
| **Demographics** |  |  |  |
| Male sex, n (%) | 29 (80.6) | 33 (76.7) | 0.681 |
| Age, median years (IQR) | 26.5 (17.0–33.5) | 20.0 (17.0–30.0) | 0.439 |
| Years in Kathmandu, median (IQR) | 9 (7–20) | 4 (2–9) | 0.134 |
| **Signs/symptoms** |  |  |  |
| Fever, n/N (%) | 33/33 (100) | 41/41 (100) | *na* |
| Headache, n/N (%) | 30/33 (90.9) | 37/41 (90.2%) | 0.923 |
| Abdominal pain, n/N (%) | 6/33 (18.2) | 17/41 (41.5%) | **0.031** |
| Typhoid triad*, n/N (%) | 5/32 (15.2) | 16/41 (39.0) | **0.024** |
| Median temperature (IQR), °C | 38.3 (37.3-39.1) | 38.4 (37.8-39.0) | 0.306 |
| Median heart rate (IQR), bpm | 100 (90–105) | 100 (100–114) | 0.260 |
| **Laboratory** |  |  |  |
| Median haemoglobin (IQR), g/dl | 14.6 (13.1–15.3) | 14.3 (12.8–15.6) | 1 |
| Median haematocrit (IQR), % | 40 (37–43) | 40 (35–43) | 0.941 |
| Median white cell count (IQR), x10^9^cells/L | 7.7 (5.4–10.0) | 5.4 (4.1–6.6) | **0.001** |
| Median platelet count (IQR), x10^9^/L | 160 (151–197) | 162 (145–199) | 0.978 |

## Supplementary Figures

**Figure S1. Individual participant ALS IgA responses to MP, LPS and flagellin before challenge with a high or low dose ST, and at typhoid diagnosis time points.** MP, membrane preparation; LPS, lipopolysaccharide. High dose, 10-50x10^3^CFU; low dose, 1-5x10^3^CFU. EU, ELISA units; TD, typhoid diagnosis.

**Figure S2. Bland-Altman plots demonstrating limits of agreement between comparisons of ALS IgA responses to MP, LPS and flagellin.** ‘*Blue’* line, regression line with 95%CI. ‘*Red dashed*’ line, limit of agreement with upper and lower 95% limits. (A) Bland-Altman (BA) plots demonstrated slight bias with higher values on average for the LPS assay than the MP assay (GMR LPS vs. MP 1.12, 95%CI 0.92−1.3)[22]. There was also a significant trend showing agreement varied across the range of assay values (regression line slope: -0.024; 95%CI -0.015 to -0.003, *p*=0.025). These observed differences are unlikely to be clinically relevant as they reveal that a one-unit change on the log scale (a 10-fold difference) results in a change in the difference in assay values of -0.024 on the log-scale (a 5% change). (B) BA plots demonstrated a strong relationship between the difference between the assays and their averages (slope: 1.28; 95%CI 1.10 to 1.45, *p*<0.001), implying a lack of agreement between the assays and large bias toward higher values for the MP assay. (C) Results for LPS compared with flagellin were similar to B (slope: 1.27, 95%CI 1.09 to 1.44, *p*<0.001).

Figure S3. Individual ALS IgA LPS and MPN responses before and up to 28 days after challenge with *Salmonella* Paratyphi A according to challenge dose and outcome. PD, paratyphoid diagnosis; nPD, no paratyphoid diagnosis. Low Dose, 0.5-1x10^3^CFU; High dose, 1-5x10^3^CFU.

Figure S4. Scatter and Bland-Altman plots demonstrating limits of agreement between comparisons of ALS IgA responses to MPN and LPS. Blue’ line, regression line with 95%CI. ‘Black dashed’ line, line of total agreement. ALS MPN and LPS assays results were slightly more divergent at higher levels, although this was not significant (Figure S3B slope: -0.03; 95%CI -0.07 to 0.01, p=0.095).

**Figure S5. ROC curves of ALS IgA responses to MPN and LPS by participants challenged with *Salmonella* Paratyphi A, using the study paratyphoid diagnosis definition as the reference standard.** The AUC values for assays were compared using the method of DeLong *et al* [33], with the null hypothesis that the difference between AUCs is 0. lgMPN, log_10-_transformed ALS IgA responses to MPN. lgLPS, log_10-_transformed ALS IgA responses to LPS.

Figure S6. Nepal ALS STARD study profile. 479 study participants excluded from the parent treatment trial were eligible for inclusion into the ALS diagnostics component. ALS performed, ALS IgA 1:4 responses to membrane preparation at day 0 results available.

Figure S7. Individual ALS responses by Nepal study participants according to blood culture result and by antigen, antibody isotype and assay dilution.

**Figure S8. Assessment of ALS IgA responses to MP and LPS at 1:2 dilution in clinical trial samples from Nepal. ‘Blue’ line, regression line with 95%CI.** (A) Scatter plot, ‘Black dashed’ line, line of total agreement. (B) Bland-Altman plot, ‘Red dashed’ lines, limit of agreement with upper and lower 95% limits; blue line slope: 0.120, 95%CI -0.096 to 0.339, *p*=0.161.

**Figure S9. Receiver-operator characteristic curves comparing the ability of ALS IgA responses to MP for the diagnosis of culture-confirmed cases of enteric fever in Patan, Nepal.** Curves were compared using the method by DeLong *et al* [33], with the null hypothesis that the difference between AUCs is 0.

**SUPPLEMENTARY REFERENCES**

1 Giavarina, D. Understanding Bland Altman analysis. *Biochem Med (Zagreb)* **25**, 141-151, doi:10.11613/BM.2015.015 (2015).

2 DeLong, E. R., DeLong, D. M. & Clarke-Pearson, D. L. Comparing the Areas under Two or More Correlated Receiver Operating Characteristic Curves: A Nonparametric Approach. *Biometrics* **44**, 837-845, doi:10.2307/2531595 (1988).
